# Increased Screen Time as a Cause of Declining Physical, Psychological Health, and Sleep Patterns: A Literary Review

**DOI:** 10.7759/cureus.30051

**Published:** 2022-10-08

**Authors:** Vaishnavi S Nakshine, Preeti Thute, Mahalaqua Nazli Khatib, Bratati Sarkar

**Affiliations:** 1 Department of Public Health Sciences, Jawaharlal Nehru Medical College, Datta Meghe Institute of Medical Sciences, Wardha, IND; 2 Department of Anatomy, Jawaharlal Nehru Medical College, Datta Meghe Institute of Medical Sciences, Wardha, IND; 3 School of Epidemiology and Public Health, Jawaharlal Nehru Medical College, Datta Meghe Institute of Medical Sciences, Wardha, IND; 4 Department of English, Jawaharlal Nehru University, Delhi, IND

**Keywords:** melatonin, vision, depression, internet, obesity, sleep quality, regulation, stress, anxiety, screen addiction

## Abstract

Dependency on digital devices resulting in an ever-increasing daily screen time has subsequently also been the cause of several adverse effects on physical and mental or psychological health. Constant exposure to devices like smartphones, personal computers, and television can severely affect mental health- increase stress and anxiety, for example, and cause various sleep issues in both children as well as adults. Risk factors for obesity and cardiovascular disorders, including hypertension, poor regulation of stress, low HDL cholesterol, and insulin resistance are among the physical health repercussions we see. The psychological health effects comprise suicidal tendencies and symptoms of depression which are associated with digital device dependency, screen-time-induced poor sleep quality, and content-influenced negativity. Oftentimes it can cause the induction of a state of hyper-arousal, increase stress hormones, desynchronize the body clock or the circadian cycle, alter brain chemistry and create a drag on mental energy and development. With a focus on brain development in children and detrimental effects in both adults and children, this research article goes on to explore the various aspects of screen addiction and excessive screen exposure.

## Introduction and background

Digital devices and online spaces, above all, are considered one of the fundamental aspects of the existence of this current generation. Rapid advancements in technology make it possible for consumers in any part of the world, regardless of age, to experience a wider variety of fast-acting stimuli that are available with similar accessibility, practically anywhere via mobile devices, enticing them to indulge in the use of screens for longer than the suggested two-three hours per day. Computers, phones, and tablets- the heralds of easy-to-use internet hosts- have seen increased purchase and usage in the present times, which has significantly reduced the distance that had once separated everything and instead turned the world into a community resembling a global village. Undoubtedly, individuals-cum-residents of this same global village have been spending an excessive amount of their time online, which has both positive as well as negative consequences, both short- and long-term. While the usage of the internet as a whole is rising, unnecessary and troublesome use of the internet has reached medically alarming levels, too. Numerous reports have highlighted the detrimental effects of its usage, such as issues that affect one's sleep, mood, and communal interactions [1]. Internet addiction (IA) is turning out to be a severe general health problem across various nations on various continents, including ours, i.e., Asia. It has been proven that one of the multiple reasons for more and more individuals being affected by depression and feeling further isolated is their addiction to mobile phones and other digital gadgets, their dependency on the presence of people on the internet, their views and values, which need not be the individual’s own. It increases dependence on validation from faceless people on the internet. It reduces the rate at which one physically interacts with others in real life, which also affects the release and maintenance of adequate doses of feel-good hormones like dopamine, serotonin, endorphins, and oxytocin, which are naturally required by all. Overuse of digital media is thought to be a significant drawback in the development of healthy psycho-physiological resilience [1]. FOMO (Fear of missing out) has also been identified as a risk factor for binge usage of the internet. The relationship between FOMO and internet usage has been extensively researched in recent studies, with younger persons more prone to the risk of the same. Along with the detrimental effect on mental health, excessive screen time is also responsible for multiple physical health deteriorations [1]. 

## Review

Effects on sleep

The use of digital media is thought to interfere with sleep in subsequent ways:

Displacing Other Activities

Screen time can replace time spent on performing physical activities, which are directly beneficial for sleep every night [1].

Time of Use

Exposure to blue and intense light in the evening and at night from self-luminous devices may prevent melatonin from being produced, alter its timings of production and retention, and thus disturb the circadian rhythm. Moreover, prolonged texting post-bedtime is likely to shorten high school kids' sleep cycles, resulting in daily drowsiness and subpar academic performance [1].

Media Type

Small touch screens, contrary to TVs, might send audible notifications during sleep time, delaying or preventing sufficient sleep. Consequently, it was observed that 18% of teenagers reported being awakened by their cell phones at least a few times throughout the night [1].

Media Content

Teenagers that use the internet more frequently have lesser durations of sleep, delayed bedtimes and wake-up times, and more daytime fatigue [2]. Those accessing social networking sites at night are more likely to sleep poorly, particularly if there is emotional involvement in the same. Additionally, engaging in an exciting task while staring at a luminous cell phone screen may enhance psychophysiological arousal, disturbing sleep [1]. The duration of time which is devoted to social media also affects how much and how well one sleeps, and good sleep is essential for academic success [3]. In 2011, Espinoza and colleagues polled 268 young adolescents specifically about social media and discovered that 37% said they had trouble sleeping due to the use of social networking sites. Adolescents use social media for 54% of their internet time. As we know, social media entails incoming alerts at all hours of the day, unlike conventional internet usage. This distinct social media characteristic is fundamental to the quality of sleep for a couple of causes [4]. First of all, because 86 percent of teenagers sleep in bedrooms with their phones nearby, frequently in their hands or shoved under their pillows, notifications at night time have the ability to disrupt sleep. They have also reported being awakened by incoming text messages and face problems in sound sleeping which are probably caused by social media [5].

Moreover, FOMO (Fear of Missing Out) is exacerbated by continual notifications, which seems to place a significant amount of pressure on people to be online all the time. Individuals mention feeling pressured, overwhelmed, and guilty if they are unable to respond to a text right away and that they face a great deal of anxiety whenever their access to messaging becomes constrained. Hence, it's likely that young people have trouble unwinding before bed because they worry about losing out on new texts or material. These special features of the usage of social media give us yet another cause to anticipate a connection between it and bad sleep [3]. A stronger correlation with specific usage of the internet at night time would suggest that exposure to digital screens before sleeping may disrupt circadian rhythms or cause sleep disruptions from notifications [5].

In contrast, a relationship between inadequate sleep and emotional involvement on the internet, specifically social media, would imply that an individual's difficulty unwinding before the night is caused by anxiety over missing out on fresh information and their inability to relax and calm down [6]. Even young children who are subjected to night-time media consumption have considerably lower sleep durations than those who are not engaged with night-time screen media [7]. The stimulating content and inhibition of natural melatonin by blue light produced via screens are two primary mechanisms underpinning the above connection. Furthermore, a persistent online presence disrupts sleep habits, that in turn influences mood [1]. Last, but not least, those who tend to spend more time online have a higher mental workload due to multitasking, increased levels of stress, and comparatively poorer quality as well as quantity of sleep, all of which are related to the deteriorating state of health [3]. *Location*

Teenage media device ownership is linked to earlier bedtimes and shorter sleep duration, relatively high bedtime resistance, with increased levels of sleep disruption, especially when the devices were kept in the bedroom [1,8].

Light Is Perceived as Electromagnetic Radiation

The occurrence of sleep during the night and the production of pineal melatonin coincides, indicating a significant link between the two processes [1]. While sleep itself is not essential for the nocturnal generation of melatonin, it is unquestionably crucial that the night-time hours be completely dark. For instance, sleep loss alone does not stop the nocturnal, circadian secretion and manufacture of melatonin [9]. Regardless of the reason for sleep loss at night, the nocturnal, circadian melatonin signal should be unaffected as long as the person is exposed to complete darkness. The term "biological night" refers to the time when melatonin is synthesized and secreted into the bloodstream and has been defined by research on humans under normal, ongoing conditions [10]. Thus, the initiation of the melatonin surge, a concomitant rise in sleep propensity, and a reduction in core body temperature marks the beginning of this biological night; the opposite occurs at the end of the biological night and sleeps [10]. The melatonin-producing pineal gland may also interpret electromagnetic radiation as light. As a result, electromagnetic radiation emitted from digital gadgets may also cause melatonin production to be hindered, which disrupts sleep [1].

Cardiovascular system

It is asserted that sedentary behavior raises the likelihood of obesity, high-density lipoprotein (HDL) dysfunction, and hypertension, the three main cardiovascular morbidity risk factors [1].

Obesity

Reduced sleep, physical inactivity, and exposure to excessive advertisements on various media have a detrimental impact on the dietary habits of the younger generation; also contributing to the correlation between screen time and obesity [11]. As far as digital media is concerned, watching television before one's bedtime is closely linked to the development of obesity during childhood and adolescence, which can further result in cardiometabolic risk [12]. In typical circumstances, a rise in satiety sensations is usually followed by an increase in plasma glucose; however, in the study, before eating, there was an increase in plasma glucose that peaked higher than it would have addressed. The findings, thus, revealed that video games are closely connected with acute stress (the fight-or-flight response). A release of glucose into the bloodstream seems to be related to this stress reaction [13].

Blood Pressure

One potential marker of future cardiovascular problems is retinal arteriolar constriction. The results show that children who spend more time outdoors tend to have bigger retinal arterioles than those who spend less time outside [14].

Cholesterol

Playing video games is the only form of sedentary activity that is associated with a decline in HDL cholesterol in obese teenagers [15,16]. Over three hours of screen usage is linked to a considerable reduction in HDL cholesterol. Mechanisms that explain this relation take into consideration the increased consumption of food advertised extensively, the decrease in food intake regulation during the time on screens, and stillness and loss of movement and physical activity. An example of this is a study that discovered that screen time leads to impairment in satiety signals through the system of mental reward, thereby directly affecting food intake [1]. 

Stress regulation

Sympathetic Arousal

Cardiovascular issues may be at risk due to chronic sympathetic arousal. Greater sympathetic arousal was observed in teenagers and youngsters who engaged in addictive online behavior. High arousal was suggested as one of the potential contributing factors to sleep disruption by researchers [1].

Cortisol

For pediatric investigations, cortisol, a hormone produced by the hypothalamic-pituitary-adrenal (HPA) axis, is a stress biomarker. Poor performance is linked to both low and elevated cortisol levels. During the nighttime, the cortisol levels are typically at their lowest. The rate of cortisol increases as the waking hours approach and undergoes a sharp spike after waking up [1]. As much as three hours per day of media usage by school-aged youngsters results in a lessened cortisol surge an hour after waking up which is detrimental [17]. Children who engage with digital media for less than three hours each day or have no digital media footprint show a regular increase in morning cortisol levels.

Insulin and Diabetes

The islets of Langerhans of the pancreas secrete the hormone insulin, which is vital for controlling metabolism and storing fat. Insulin resistance is a condition that occurs when cells are unable to use insulin effectively. It makes an essential contribution to the pathophysiology of diabetes and increases the risk of developing cardiovascular disease. According to some research, watching television, playing video games, or using the computer for more than an additional hour a day can result in a 5% reduction in insulin sensitivity. Other research linked as little as two hours of screen time per day to aberrant insulin levels [1].

Vision

Constantly staring at a PC screen can result in headaches, eye strain, impaired vision, dry eyes, and irritation. Such symptoms may be brought on by glare, inadequate illumination, or a wrong viewing angle. According to research, being in an outside environment stimulates the release of dopamine from the retina, which prevents myopia, or near-sightedness, from developing [18]. As a result, children who spend fewer hours outside have a higher chance of developing the same. Additionally, spending time outside can lessen and largely eliminate the factors that contribute to the development of myopia, such as prolonged close work or screen viewing. The participating young subjects who have been playing video games for more than 30 minutes nearly on a day-to-day basis reported headaches, vertigo, and eye strain. The dominant eye primarily experienced transient diplopia and refractive problems (such as short-sightedness), ultimately leading to vision loss [1].

Orthopedics

Sedentary habits, or sitting activities that don't involve exercise, can have a significant impact on one's joints and bones. It is argued that screen usage, especially on small-screen mobile devices, affects posture and causes musculoskeletal strain and pain sensations. Similar symptoms can be brought on by the frequent, repeated wrist and arm movements and head inclination typical of playing video games. Bone mineral density is found to be negatively correlated with boys' video gaming play time. The mineral composition of the femoral and spinal bones is inversely correlated with girls' screen time [1].

Depression and suicidal behavior

Depression, mainly social media-induced depression is a growing concern, particularly among today’s generation [19]. Those not aware of the usage of social media effectively can easily get trapped in a pattern of jealousy, envy, self-doubt, and poor self-esteem [20]. It is well known that sleep disturbance symptoms appear before symptoms of depression and suicidal thoughts. Therefore, it is proposed that a mediating element connecting night-time screen use to depressive symptoms and suicidal thoughts in adolescents is the lack of sleep [21]. The researchers further note that dependence on smartphones, frequent messaging, and protracted fear about not receiving back messages, particularly before bedtime, are likely associated with mood swings, suicidal thoughts, and self-injury. 

ADHD (attention deficit hyperactivity disorder)

Children in today's society may oftentimes exhibit different symptoms of ADHD, such as inattentiveness, hyperactivity, and impulsivity. This type of conduct is known as ADHD-related behavior and can be connected to one's screen time [22]. It has also been stated that excessive digital device usage is prevalent among younger children and teenagers who have either been already diagnosed with ADHD or who are regarded to be dealing with attention issues or impulsivity [23].

Addictive screen time behavior

While a substantial percentage of men seem to exhibit video game addiction, women on the other hand are primarily focused on social networking [1].

Neuropsychological Effects

Numerous investigations so far have concluded that any form of addiction to the internet causes structural changes in the brain, specifically in its frontal lobe. The ability to filter out irrelevant information and a reduced capacity for coping with demanding and complex tasks are related to such structural alterations. The frontal lobe is majorly concerned with controlling overly assertive and wrong, miscellaneous behaviors, as well as adjusting to environmental change [1]. Other research has shown that control over one’s emotions, visible discord during the decision-making process, and compulsively repetitive behaviors are linked to damaged white matter. Studies have examined the impact of screen multitasking when continuous attention across media devices becomes a substitute for "real world" behavior [4]. The anterior cingulate cortex, which is connected to cognitive control of performance and socioemotional regulation, is found to have less grey matter in college students with high multitasking scores. Poor performances have been recorded in college students who have been switching between tasks, working memory, and filtering through tests [24]. Another study on the same group found links between less grey matter and poorer conflict detection of conflict, increased neuroticism and impulsivity, poorer control over behavior that are goal-oriented, and increased conduct motivated by sensations [1].

Behavioral and Societal Aspects of Social Media Usage in Digital Spaces

This cohort is characterized by increased attentional lapses that have been self-reported regularly, a non-deliberate occasion of mental wandering, which is consistent with the result that grey matter decreases in heavy media multitasking young adults [25]. The main symptom of ADHD in university students is non-deliberate mind-wandering, which is linked to decreased levels of mindfulness and a higher prevalence of non-adaptive or negative thought patterns. Teenagers who are hooked to the internet and exhibit stronger depressive, hostile and ADHD-related symptoms are found to have non-adaptive/negative thinking patterns [26]. Therefore, it would seem that media addiction is correlated with increased mental wandering. Individuals who multitask frequently and are screen addicts were also discovered to have lower levels of social support and peer support or attachment to their families and relations. As a result, their level of life contentment is lowered. Teenagers are moving away from face-to-face connection, which is limiting offline social support even though it has increasingly been linked to positive social well-being [27]. They are more likely to get caught up in a dangerous cycle of continued usage of the internet and social networking websites in an effort to rekindle to social support that they crave when facing obstacles. However, the guise of support that individuals can receive online aids in sustaining their repetitive Internet usage even more [28]. On the other side, social support that is unrelated to screens may reduce internet addiction. A lack of societal support and increased mind wandering are likely to facilitate social functioning and raise the likelihood of more profound sadness, loneliness, and isolation, which may further enhance addictive behavior [29,30].

Additionally, it is highlighted that social support, attachment to materialistic or figurative objects, mindfulness of one’s surroundings and the feeling of others, and degree of life satisfaction are all psychological and social aspects that are adversely impacted by compulsive screen use and are essential to an individual's resilience in the face of adversities in life [31]. Furthermore, behavior resulting in incidents of cyberbullying and the social components of addicted internet use appears to be related [1]. Women in their early adolescence and early years of adulthood are more likely than men to spend more time on social media, be subjected to higher risks of cyberbullying, and experience detrimental mental health issues, the aforementioned are proving to be consistent with the recent epidemiologic trends illustrating that young females, in particular, are more likely to experience an increase in symptoms of depression, self-harm tendencies, and suicidal thoughts [32].

Radiation

With children increasingly using wireless gadgets, worries about their susceptibility to radio-frequency electromagnetic radiation (RF-EMR) fields are growing. Kids' growing neurological systems are thought to be quite susceptible to RF-EMR fields, making them possibly more vulnerable. Moreover, compared to the size of their head, more RF-EMR can penetrate their brain tissue because it is better conductive. They will also be exposed to RF fields for more years than grown-ups [1].

Infertility

Infertility is a common condition that affects 7% of men and 11% of women in the U.S. [1]. There is a necessity to establish the links between environmental exposures and sperm quality indicators are given that there is evidence of a deterioration in semen quality in recent years [33]. Exposure to smartphones has been linked to reduced sperm viability and motility. Studies on experimental animals and people examined how RF-EMR also affected male reproductive function. On biological tissue, RF-EMR might have thermal and non-thermal effects both. Reactive oxygen species (ROS) may be produced more frequently as a result of nonthermal interactions, which may cause DNA damage. A little amount of ROS has a crucial functional role in the acrosome response, binding to the oocyte, as well as sperm capacitation. Since phones are frequently kept in pant pockets close to the reproductive organs, thermal impacts potentially raise the temperature of the testes, hampering spermatogenesis and sperm production [33]. Mobile phones are found to be a potential contributing factor in light of newly discovered evidence of a deterioration in the quality of male sperm. According to the findings of these studies, using a cell phone or a laptop, or a tablet when exposed to RF-EMR increases one's risk of developing cancer as well [1].

A decline in academic performance

The findings in a cohort study indicate that overall screen time, and the duration spent using a smartphone, are both strongly correlated with the academic stress score and increases the likelihood of abnormal academic stress. One explanation might be the use of cell phones and other electronic devices for homework. This is corroborated by a research study that found that children utilize smartphones and other devices mostly to complete their homework. Hence, the amount of time spent using a device may partly reflect how much schoolwork pupils have to do and the stress that comes with it [34]. Utilizing electronic devices for social interaction and entertainment may be another factor. More than 30% of teenagers, according to one research, use cell phones and other gadgets for social interaction. Spending a lot of time on social activities and entertainment might interfere with study time, hindering students' academic achievement and raising stress levels [35]. In the meantime, a bidirectional association has also been established between screen time and academic stress because of the slow progress of studies, which could lead to additional screen time. Furthermore, using a smartphone for social media and amusement might make it difficult for students to concentrate on their work, which could result in unsatisfactory academic results. A rise in academic stress could also eventually be triggered by inferior academic achievements [36]. The range of digital media devices is expanding rapidly, and improving digital media provides society with a more vibrant and quick-paced digital world. Children and teenagers in particular seem to adapt to modern technologies quickly. But, a growing body of literature associates excessive screen time with physical, psychological, social, and neurological adverse health consequences. Developmental, pornographic exposure and learning effects are additional effects of screen time that require further in-depth analysis and are beyond the scope of this review article. Until very recently, the majority of the literature on children and adolescents use of digital media focused on TV and computers. However, as people seem to utilize mobiles increasingly, studies are shifting their focus accordingly [1]. Even though the Internet continues to be a powerful and uplifting force in the lives of many, a percentage of users may develop an addiction. Due to their growing online usage and heightened susceptibility to the emergence of addictive behaviors, adolescents are a noteworthy worry in this regard [37]. Undoubtedly one of the most difficult problems facing the social sciences to this date is the increase in smartphone and social media use. Popular science books and news reports on the psychological effects of social media use are in high demand, and many of these works paint a very ominous picture: social media is often said to have a negative impact on people's lives and societies [3].

Discussion

**Figure 1:**
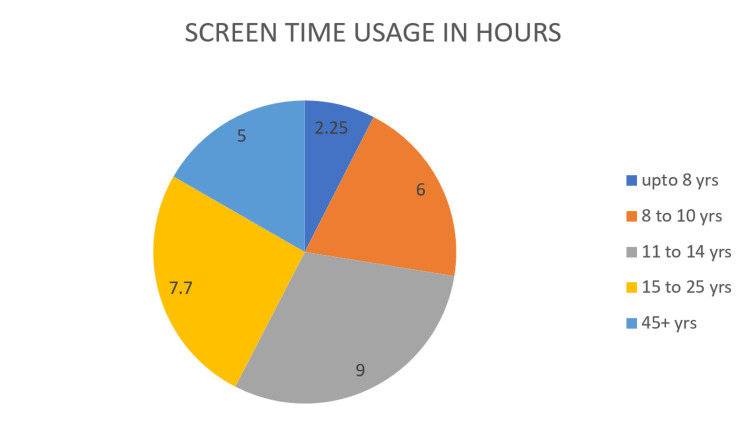
Use of screen time in hours

**Figure 2:**
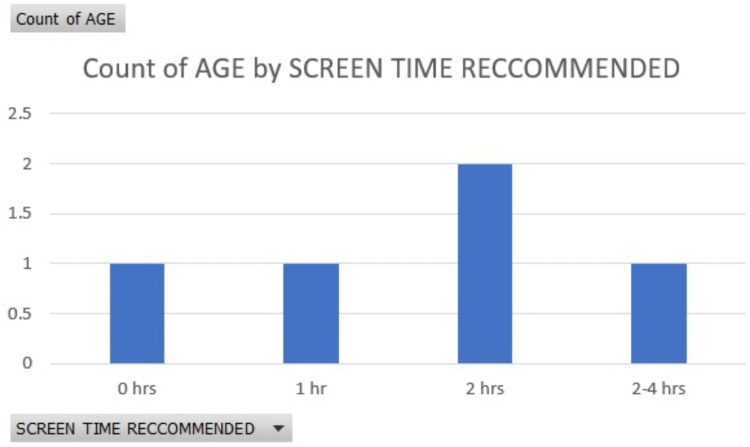
screen time recommended as per age This figure has been taken from an open-access journal under a CC-BY license. Source: American Academy of Child and Adolescent Psychiatry [38].

Excessive Internet use has negative effects that are obvious and detrimental, ranging from sleep deprivation, increased depression, and skipping school to family conflict. Comorbid depression, anxiety, ADHD, and other substance-use disorders are widespread. Various markers of individual personality, such as impulsivity, aggressiveness, sensation seeking, family conflicts, as well as inadequate parental supervision, also especially belonging to the male gender have proven to be additional risk factors [8]. Non-adaptive or negative thought patterns, a lower sense of fulfillment in life, and a propensity for health concerns throughout maturity are several other characteristics that one may notice appearing. The concluding results present that a lack of physical, real-life interaction, extensive multitasking, increased usage of social media websites, and interactive screen time through addictive web interfaces- mostly through the regular usage of video games that require online multiplayer interactions, which do not allow the players to pause the games or exit the rounds- all of these play a significant role in determining the emotional, psychological and physiological impacts on the population. According to research from the Centers for Disease Control and Prevention (Figure 1), children aged 8 to 10 spend six hours per day using screens, children aged 11 to 14 spend nine hours, and teenagers aged 15 to 25 spend seven and a half hours per day using screens (including television). The chart below (Figure 2) shows screen time recommendations by age, from infants to adults based on the study by the American academy of child and adolescent psychiatry.

Overusing digital screens during one's adolescence and young adulthood may result in their mind relating to outside stimuli rather readily and cause a lack of attention. Internal triggers such as unhelpful or negative thoughts and feelings of lower satisfaction levels regarding one's life can also be accompanied by an onset of health problems in adulthood, such as cardiovascular disease and infertility. It may cause one's stress to increase up to levels that would turn difficult for one to handle. These ailments can result in unhealthy coping mechanisms, which eventually might increase the likelihood of sadness, depression, and anxiety in one's later years. One can successfully deal with difficult life situations if they have a strong sense of personal resilience. Physical fitness, societal support, forms of attachment, the mindfulness towards issues, and the degree of life pleasure, both temporary and permanent, are some of the crucial components of resilience, which is a dynamic psychophysiological construct that can stand jeopardized by excessive screen time and digital footprints. Therefore, it indicates that individuals who use digital media excessively and compulsively have been compromising their ability to build solid psychophysiological foundations, which are essential for the development of resilient people in the future [1]. Moreover, the COVID-19 pandemic has significantly expanded the use of video conferencing. People of all ages are using video conferencing apps for regular social activities like meetings, birthdays, exercises, and so on due to social distance laws and travel limits. Such video conferencing apps have also made body dysmorphic disorder worsen and are very concerning in the long run. Patients with pre-existing BDD spend too much time and money on a variety of treatments to correct their perceived flaws [39]. Hence, excessive screen time, be it recreational or non-recreational, can result in a plethora of disadvantages and harm individuals in ways that could perhaps cause irreversible damage to them throughout their entire lifetime. According to numerous studies, time spent on screens irrefutably proves to be more harmful instead of beneficial. With the advancement of technology, it is to be acknowledged that individuals in the contemporary era are extensively involved with the digital world with or without their consent, with regard to their school and university studies or work responsibilities. It is thus necessary for one to recognize the opportunities and obstacles that social media and digital spaces offer and navigate them accordingly. 

## Conclusions

This review article studied the relationships between screen time and digital device usage, precisely during the night times, the quality of sleep, anxiety causes, feelings of depression, and issues related to self-esteem, as well as physical effects in individuals. Results also show that exposure to mobile phones has a deleterious impact on the viability and motility of sperm. 

A strategy that would prohibit digital media usage would be ineffective given how crucial it is for one to be involved in digital spaces, specifically social media norms. That being said, in the context of numerous initiatives aimed at addressing the societal, environmental as well as economic factors that support the betterment of one's family and foster resilience in the youth, who could also readily benefit from proven systemic and specifically conducted individual interventions to assist them in navigating through the hurdles brought on by the usage of cell phones and social media, thus, protecting themselves from harm, and further using it in a certain way that maintains their emotional wellbeing above all. However, from the perspective of public health, screen time in general, and the time spent using a smartphone, should be limited in order to lessen and prevent several linked health issues.
